# Total minimally invasive McKeown esophagectomy in an esophageal cancer patient with situs inversus totalis: A case report

**DOI:** 10.1111/1759-7714.13723

**Published:** 2020-11-05

**Authors:** Chu‐Long Xie, Jing‐Sheng Cai, Zi‐Hui Tan, Jie Yang, Hao‐Xian Yang

**Affiliations:** ^1^ Department of Thoracic Surgery Sun Yat‐sen University Cancer Center Guangzhou China; ^2^ State Key Laboratory of Oncology in South China, Collaborative Innovation Center for Cancer Medicine Sun Yat‐sen University Cancer Center Guangzhou China

**Keywords:** Esophageal cancer, situs inversus totalis, total minimally invasive esophagectomy

## Abstract

Situs inversus totalis (SIT) is an extremely rare anomaly characterized by a left‐to‐right reversal of all the thoracic and abdominal organs. Only 11 cases of esophageal cancer with SIT have been reported worldwide, most of which underwent hybrid minimally invasive esophagectomy (MIE) but not total MIE. Here, we report a case of esophageal cancer with SIT successfully treated by total MIE, with a right lateral‐prone position adopted during the thoracic procedure. The relevant literature is also discussed and reviewed.

## Graphical abstract

Situs inversus totalis (SIT) is an extremely rare anomaly characterized by a left‐to‐right reversal of all the thoracic and abdominal organs. Only 11 cases of esophageal cancer with SIT have been reported worldwide, most of which underwent hybrid minimally invasive esophagectomy (MIE) but not total MIE. Here we report a case of esophageal cancer with SIT successfully treated by total MIE, with right lateral‐prone position adopted during the thoracic procedure.

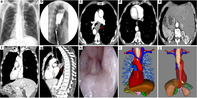

## Introduction

Situs inversus totalis (SIT), with an incidence of 1/8000 to 1/25000 live births, is a rare anomaly that refers to a mirror image reversal of the thoracic and abdominal organs.^1^ To the best of our knowledge, there have only been 11 cases of esophagectomies for esophageal cancer with SIT reported worldwide so far, most of which underwent hybrid minimally invasive esophagectomy (MIE) and a right decubitus or prone position was adopted during the thoracic procedure.^2^, ^3^, ^4^, ^5^, ^6^, ^7^, ^8^, ^9^, ^10^, ^11^ Here, we report a case of esophageal cancer with SIT successfully treated by total MIE, with a right lateral‐prone position adopted during the thoracic procedure.

### Case report

**Figure 1:**
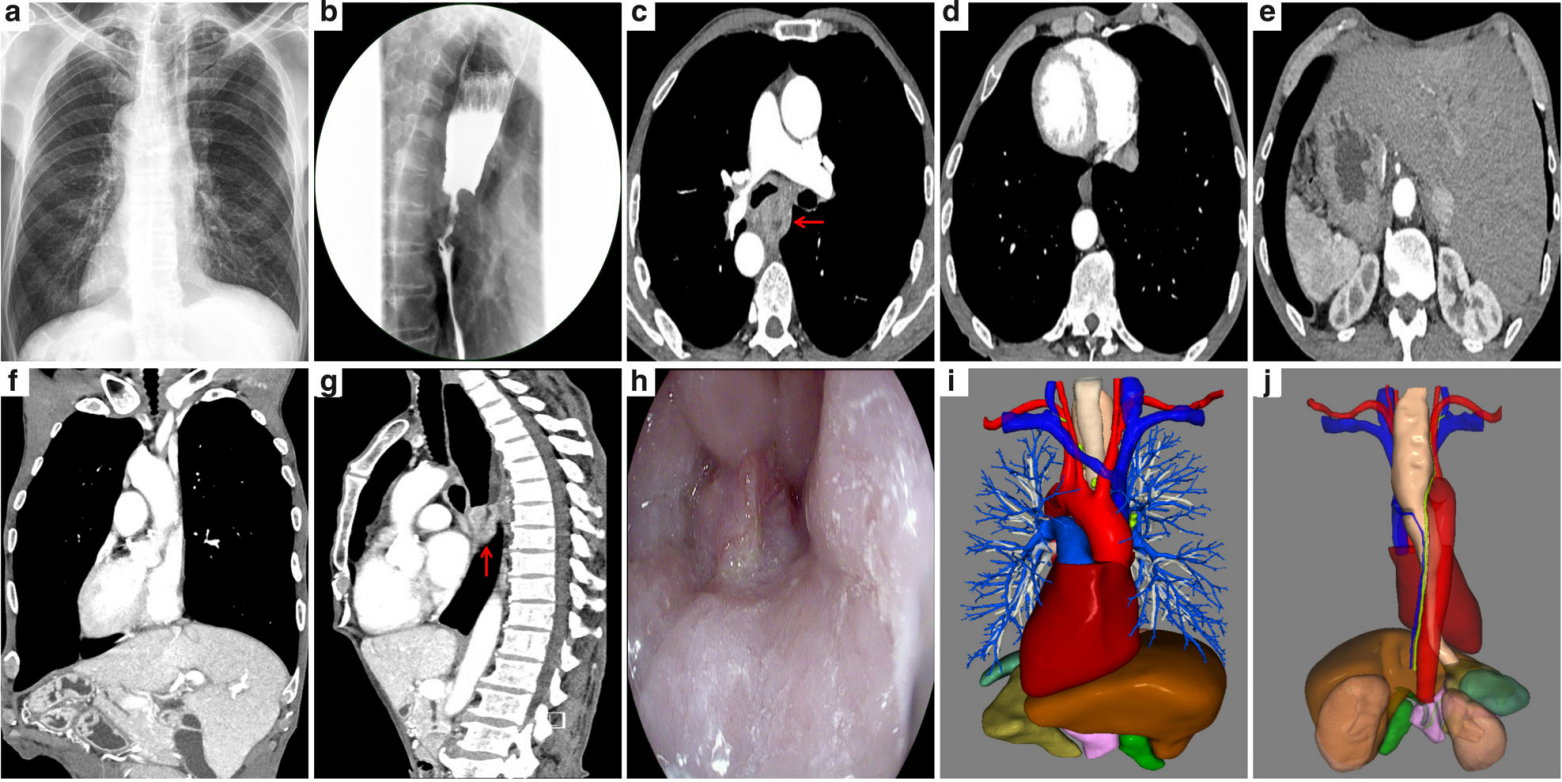
Preoperative imaging examination confirmed the presence of SIT and middle third esophageal cancer. (**a**) Chest X‐ray showing dextrocardia and a right aortic arch; (**b**) Barium swallow showing the tumor in the middle third esophagus; Cross section (**c**) and sagittal section (**g**) of CT showing the tumor (arrow); Chest CT (**d**), Abdominal CT (**e**) and coronal section of CT (**f**) showing SIT; (**h**) Endoscopic ultrasound revealed an ulcerated esophageal tumor; Anterior (**i**) and posterior views (**j**) of 3‐D images demonstrating the surrounding structures around the esophagus.

A 66‐year‐old male presented with a three‐month history of progressive dysphagia. The chest X‐rays, barium swallow and computed tomography (CT) confirmed the presence of SIT and middle third esophageal cancer (Fig 1a–g). Endoscopic ultrasound revealed an ulcerated tumor diagnosed as squamous cell carcinoma by biopsy (Fig 1h). Three‐dimensional (3‐D) images were constructed to demonstrate the surrounding structures around the esophagus (Fig 1i, j). The patient was diagnosed with middle third esophageal cancer (cT2N2M0, Stage III) and total minimally invasive McKeown esophagectomy was performed because the patient declined neoadjuvant chemoradiotherapy.

**Figure 2:**
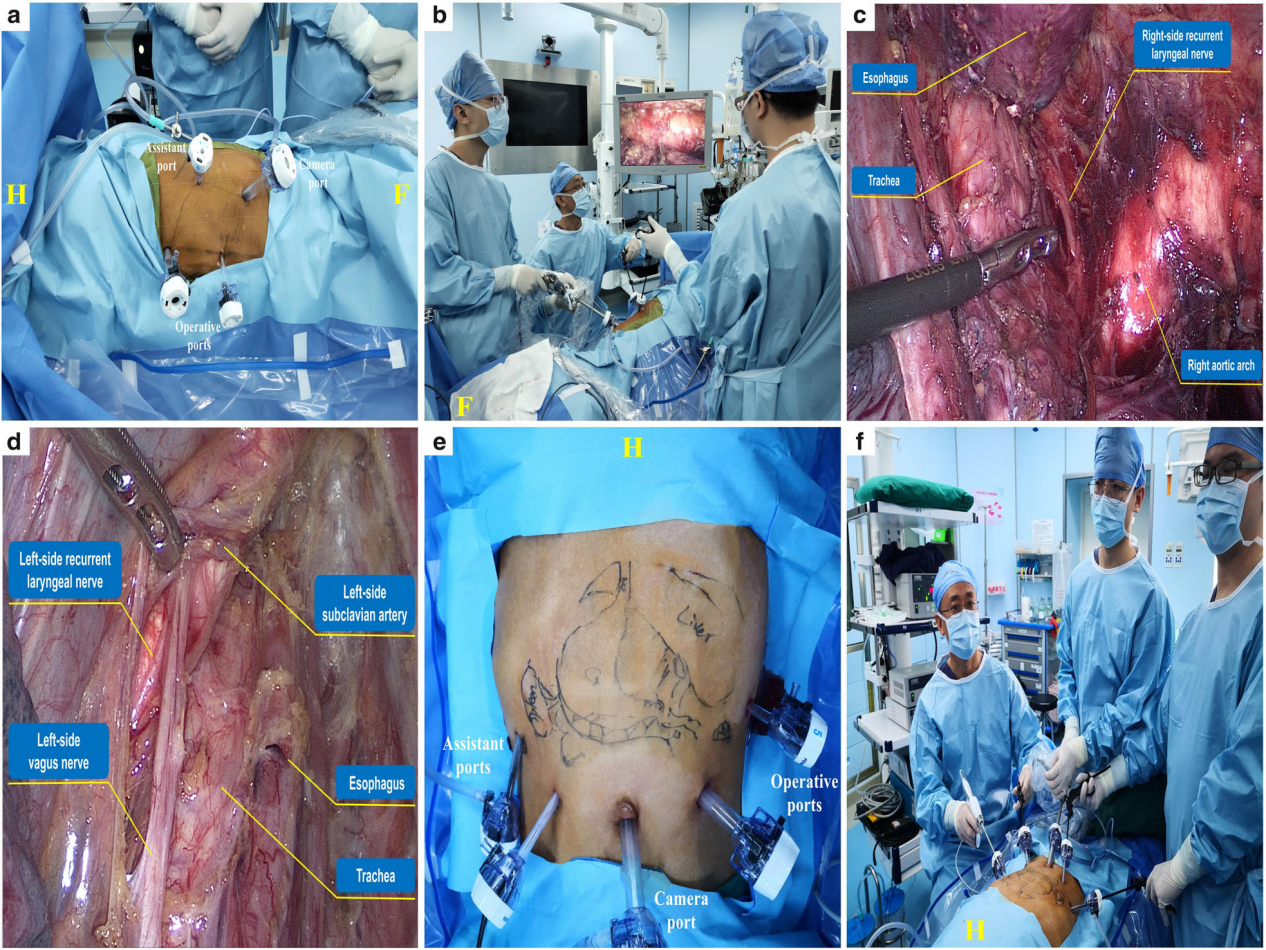
Surgical views. (**a**) Position and ports in thoracoscopic procedure; (**b**) Surgeon's position during the thoracic procedure; (**c**) Anatomic structures of the right‐side (**c**) and left‐side (**d**) recurrent laryngeal nerve; (**e**) Position and ports in laparoscopic procedure; (**f**) Surgeon's position during the laparoscopic procedure. H, head; F, foot.

Double lumen trachea insertion and single‐lung ventilation were applied for anesthesia. The operation was divided into three phases: (i) the thoracoscopic operation phase to mobilize the esophagus and dissect the mediastinal lymph nodes; (ii) the laparoscopic operation phase to mobilize the stomach, dissect abdominal lymph nodes and create a gastric conduit; (iii) the cervical operation phase to perform anastomosis and dissect cervical lymph nodes through a right (not left) neck incision. CO_2_ insufflation was applied in both phase 1 and phase 2 with a pressure of 6 and 12 mmHg, respectively. In phase 1, the patient was placed in the right lateral‐prone position using four ports (Fig 2a). The camera port was set in the seventh intercostal space at the posterior axillary line. Two operation ports were set at the third intercostal space (left hand of the surgeon) and fifth intercostal space (right hand of the surgeon), respectively, both of which were at the anterior axillary line. Another port was set in the sixth intercostal space anterior to the tip of the scapula to assist the physician. The surgeon performed the surgery in a sitting position at the ventral side of the patient (Fig 2b). The thoracic duct was dissected in a gross specimen with the esophagus and the paraesophageal tissues and recurrent laryngeal nerve on both sides were preserved (Fig 2c, d). In phase 2, the patient was placed in the supine position with legs split apart (Fig 2e). Five ports were placed in an arched shape on the upper abdomen, consisting of a camera port below the umbilicus, two operation ports on the left midclavicular line 3 cm above the umbilicus and left anterior axillary line below the costal arch, respectively, and two assistant ports on the right midclavicular line 3 cm above the umbilicus and right anterior axillary line below the costal arch, respectively. The surgeon sat at the left side of the patient while the camera‐holding assistant stood between the legs of the patient (Fig 2f). A gastric conduit was made via a 4 cm vertical median incision in the upper abdomen after the laparoscopic operation was completed. In phase 3, the gastric conduit was pulled upward to the right neck incision via the retrosternal space, and cervical esophago‐gastric end‐to‐side anastomosis was performed with the use of a circular stapler.

**Figure 3:**
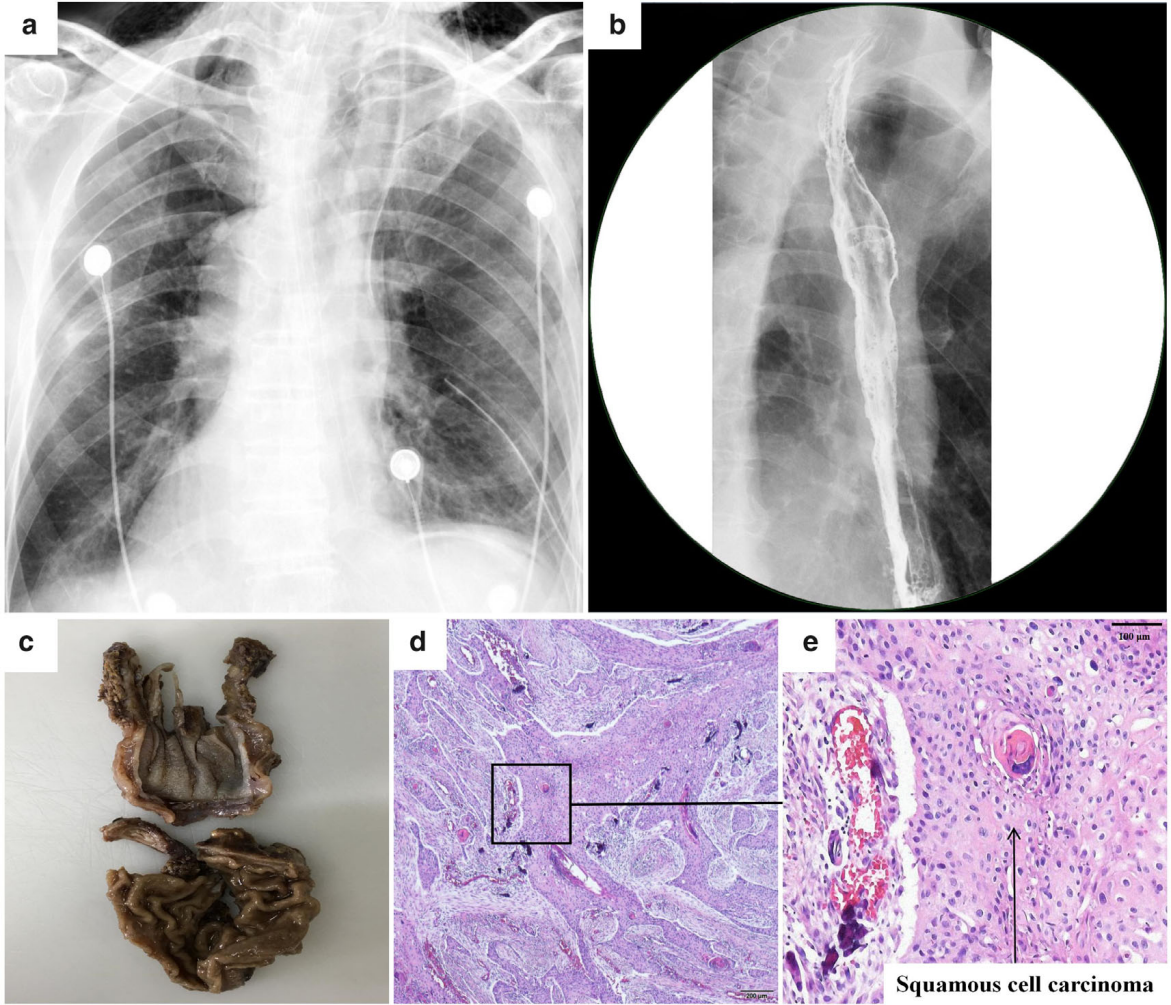
Postoperative imaging examination and histopathology. (**a**) Chest X‐ray of postoperative day 1; (**b**) Barium swallow of postoperative day 16; (**c**) Gross tumor specimen; (**d**, **e**) Moderately differentiated squamous cell carcinoma confirmed by histopathology.

A total of 48 lymph nodes were harvested during surgery. Total operative time from incision to closure was 480 minutes and the estimated blood loss was 80 mL. Postoperative examination was normal (Fig 3a, b), and the patient was discharged on postoperative day 7 without any remarkable complications. Histopathology of the resected specimen confirmed squamous cell carcinoma with pT3N1M0, stage IIIB disease (Fig 3c–e).

## Discussion

Situs inversus totalis (SIT) is a rare congenital anomaly of unknown etiology, and most people with SIT are asymptomatic and unaware of their unusual anatomy until they present for imaging examination of unrelated diseases. To the best of our knowledge, only 11 cases of esophagectomy for esophageal cancer with SIT have been previously reported globally (Table 1).^2^, ^3^, ^4^, ^5^, ^6^, ^7^, ^8^, ^9^, ^10^, ^11^

**Table 1:** Summary of 12 cases of esophagectomy for esophageal cancer with situs inversus totalis

Case	Age (years)	Sex	Comorbidity	Operative procedure	Position in thoracic surgery	Operative time (minutes)	Blood loss (mL)	Perioperative complications	Hospitalization days
Yoshida *et al*.^2^	57	M	None	Thoracoscopic + hand‐assisted laparoscopic surgery	Right decubitus	540	340	None	Not described
Mimae *et al*.^3^	57	M	None	Thoracotomy + laparotomy	Right decubitus	512	585	None	16
Aoki *et al*.^4^	53	M	None	Thoracotomy + laparotomy	Right decubitus	463	762	None	18
Yagi *et al*.^5^	73	M	None	Thoracoscopic + hand‐assisted laparoscopic surgery	Right decubitus	390	130	None	Not described
Peel & Darling^6^	67	M	Kartagener syndrome	Thoracoscopic + laparoscopic surgery	Right decubitus	Not described	Not described	Not described	Not described
Ujiie *et al*.^7^	63	M	None	Thoracoscopic + hand‐assisted laparoscopic surgery	Right decubitus	621	310	None	17
Chinusamy *et al*.^8^	62	M	None	Thoracoscopic + laparoscopic surgery	Prone	286	Not described	None	11
Hosoda *et al*.^9^	78	M	None	Thoracoscopic surgery + laparotomy	Right semiprone	861	978	Right recurrent laryngeal nerve palsy	30
Nakano *et al*. (1)^10^	82	M	None	Thoracoscopic + hand‐assisted laparoscopic surgery	Prone	661	157	None	34
Nakano *et al*. (2)^10^	66	M	Intestinal malrotation; polysplenia	Thoracoscopic surgery + laparotomy	Prone	637	210	None	17
Feng *et al*.^11^	54	M	None	Thoracotomy + laparotomy	Right decubitus	Not described	Not described	None	10
Our case	66	M	None	Thoracoscopic + laparoscopic surgery	Right lateral‐prone	480	80	None	9

Along with advances in endoscopic technology, MIE has gradually emerged as an effective alternative to open surgery.^12^ MIE mainly consists of total MIE (combined thoracoscopic‐laparoscopic esophagectomy) and hybrid MIE (thoracoscopic‐assisted esophagectomy or laparoscopic‐assisted esophagectomy). Most reported cases of esophageal cancer with SIT underwent hybrid MIE and the right decubitus position was adopted during the thoracic procedure. Here, we report a case of esophageal cancer with SIT successfully treated by total MIE, with the right lateral‐prone position adopted during the thoracic procedure. Compared with the lateral decubitus position, the lateral‐prone position combined the advantages of both the lateral decubitus position (allowing quick conversion to open surgery) and prone position (providing a well‐exposed operative field for esophagus), which was also less physically demanding for the surgeon to operate in a sitting position (Fig 2b, f). Some retrospective studies have suggested that MIE in the lateral‐prone position could be a reliable approach for thoroughly resecting thoracic esophagus and dissecting total mediastinal lymph nodes.^13^, ^14^

Surgical procedures in esophageal cancer patients with SIT might technically be more challenging due to two main difficulties during surgery. With regard to our report, the first challenge was the fact that the surgeon found it difficult to localize and identify the recurrent laryngeal nerves because the left‐side recurrent laryngeal nerve looped under the left‐side subclavian artery while the right‐side recurrent laryngeal nerve looped under the right aortic arch. The second came from the fact that the right‐handed surgeon felt more impairment when dissecting with his left hand for some procedures, such as mobilizing and dissecting the tissues in the outlet of the thoracic cavity. However, the surgery could still be performed as well as the routine procedures after careful recognition and rapid adaptation of the mirror‐image anatomy. In addition, preoperative three‐dimensional (3D) image reconstruction was a helpful adjunct as it provided the most graphic representation of the orientation of organs, especially for patients with SIT.

Taken together, here we present a rare case of esophageal cancer with SIT that was successfully treated by total MIE. Although the surgical procedures of esophageal cancer with SIT are technically more challenging, the surgical procedure itself does not differ from routine surgery after careful recognition of the mirror‐image anatomy. Total MIE should therefore be considered as one of the feasible and safe procedures for esophageal cancer with SIT.

## Disclosure

The authors have no conflict of interest to disclose.
